# Physical Health Needs of Older Adults with Substance Use Disorder

**DOI:** 10.1192/bjo.2026.11396

**Published:** 2026-06-30

**Authors:** Sururat Ibrahim, Tracey Myton

**Affiliations:** 1 Lancashire and South Cumbria NHS Foundation Trust, Preston, United Kingdom; 2 Greater Manchester Mental Health NHS Foundation Trust, Prestwich, United Kingdom

## Abstract

**Aims:**

To investigate substance use patterns among older adults engaged with Bolton Addiction Service, with a particular focus on their physical health needs and service provision.

**Objectives:**

- To determine the prevalence of physicalcomorbidities among this population.
- To evaluate the frequency and adequacy of physical health monitoring provided to older service users.
- To assess whether current service provision meets the specific needs of older adults and to develop recommendations for service improvement.

**Methods:**

We employed a retrospective design, drawing on clinical records from the Bolton Addiction Service (Paris and GM care records). 65 years was the operational cut-off for defining “old age”. 33 records were accessed for their:

–Sociodemographic characteristics and social circumstances.

–Substance use and treatment history.

–Clinical profile: presence of comorbid medical conditions and concurrent medications.

–Investigations: whether any diagnostic or monitoring investigations were recorded, and the time elapsed since the most recent investigation.

–Cognitive status: evidence of cognitive decline documented in clinical notes.

**Results:**

The project highlighted significant physical and social vulnerabilities among older adults receiving treatment for substance misuse, with over halfliving alone and almost all of them relying on pension or benefits.

- Opioid misuse is the predominant issue(73%), with 79% of these having a history of injecting, although only a small proportion (8%) continue to inject. 91.6% of those with opioid misuse are on methadone.
- Two thirds have been in treatment for more than five years,many(39%) for over two decades. Those with shorter treatment histories are largely individuals with alcohol misuse, who receive psychosocial interventions rather than medication.
- Nearly all patients have multiple medical comorbidities, most commonly cardiovascular disease, and all are prescribed several medications.
- Cognitive impairment is documented in one fifth of the cohort, including three cases of confirmed dementia.
- Despite this high level of clinical complexity, more than half have not received a medical review in the past year, eight had no physical health investigations, and only one patient underwent an ECG.

**Conclusion:**

Older adults with substance misuse have substantial physical health needs that are not fully met by the current service. Enhancing the frequency of medical reviews and physical health investigations, particularly ECG monitoring, is recommended. It was also suggested that doctors include discussion about methadone dose reduction or change to buprenorphine as part of older adults’ medical reviews.

